# Supplementary material for: Presence of CD44v9-Expressing Cancer Stem Cells in Circulating Tumor Cells and Effects of Carcinoembryonic Antigen Levels on the Prognosis of Colorectal Cancer
Source: Cancers (Basel). 2024 Apr 19;16(8):1556. doi: 10.3390/cancers16081556 (PMC11048819; doi:10.3390/cancers16081556)
Supplement: Supplementary file 1 [file cancers-16-01556-s001.zip › supplementary figure PDF.pdf]

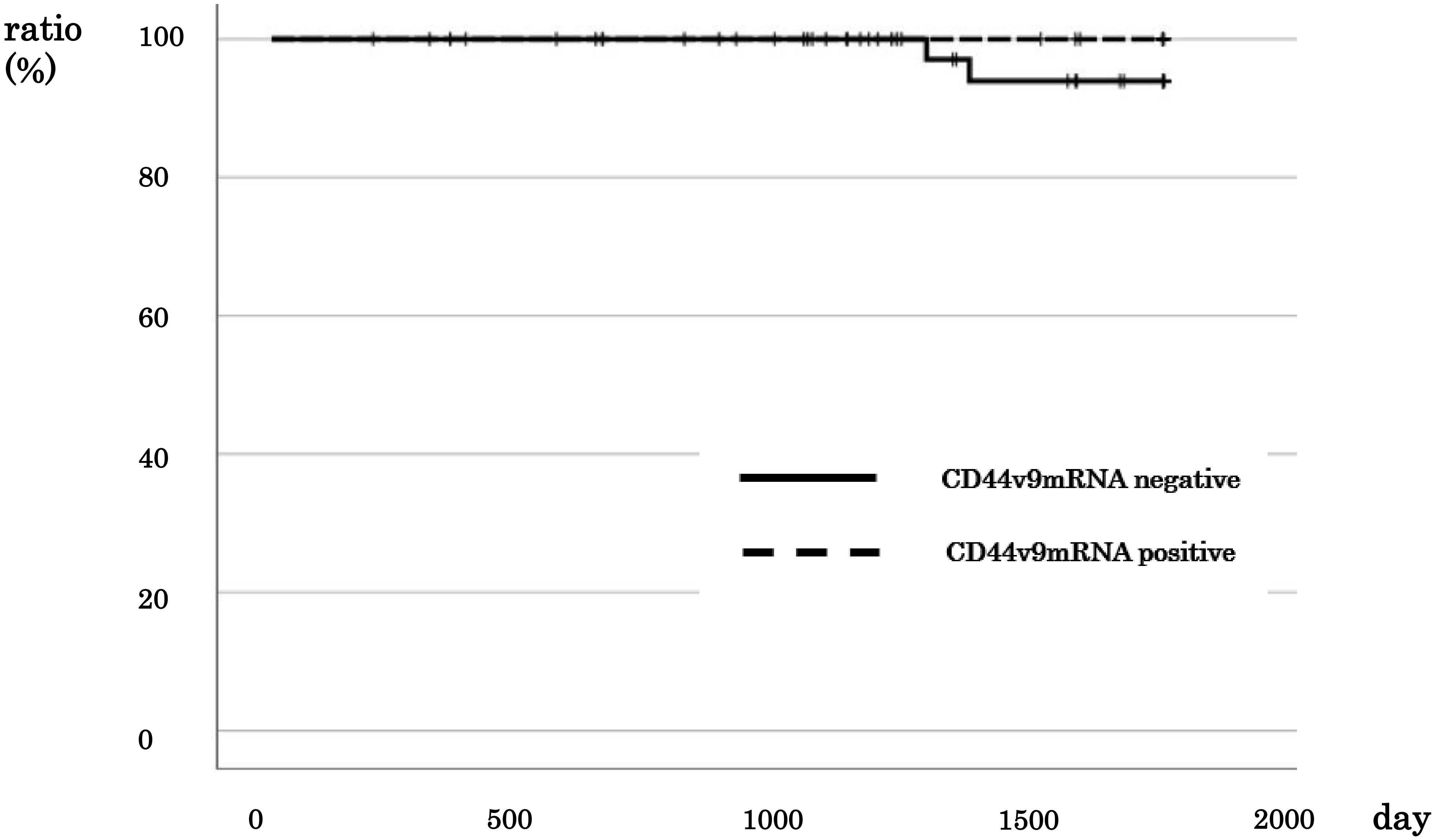

Figure S1a. Relationship between *CD44v9* mRNA expression and survival rate in patients with stage I of colorectal cancer.

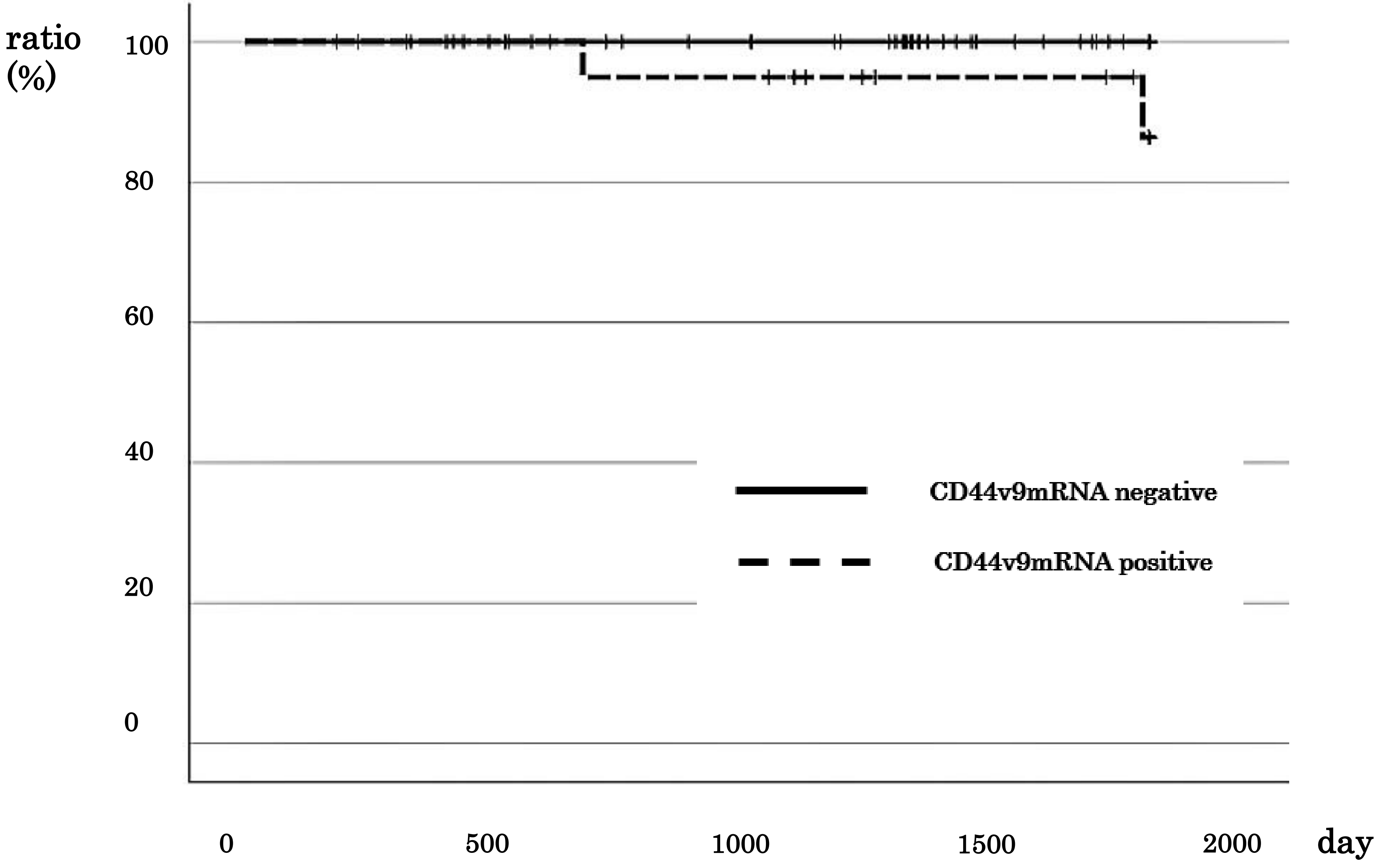

Figure S1b. Relationship between *CD44v9* mRNA expression and survival rate in patients with stage II of colorectal cancer.

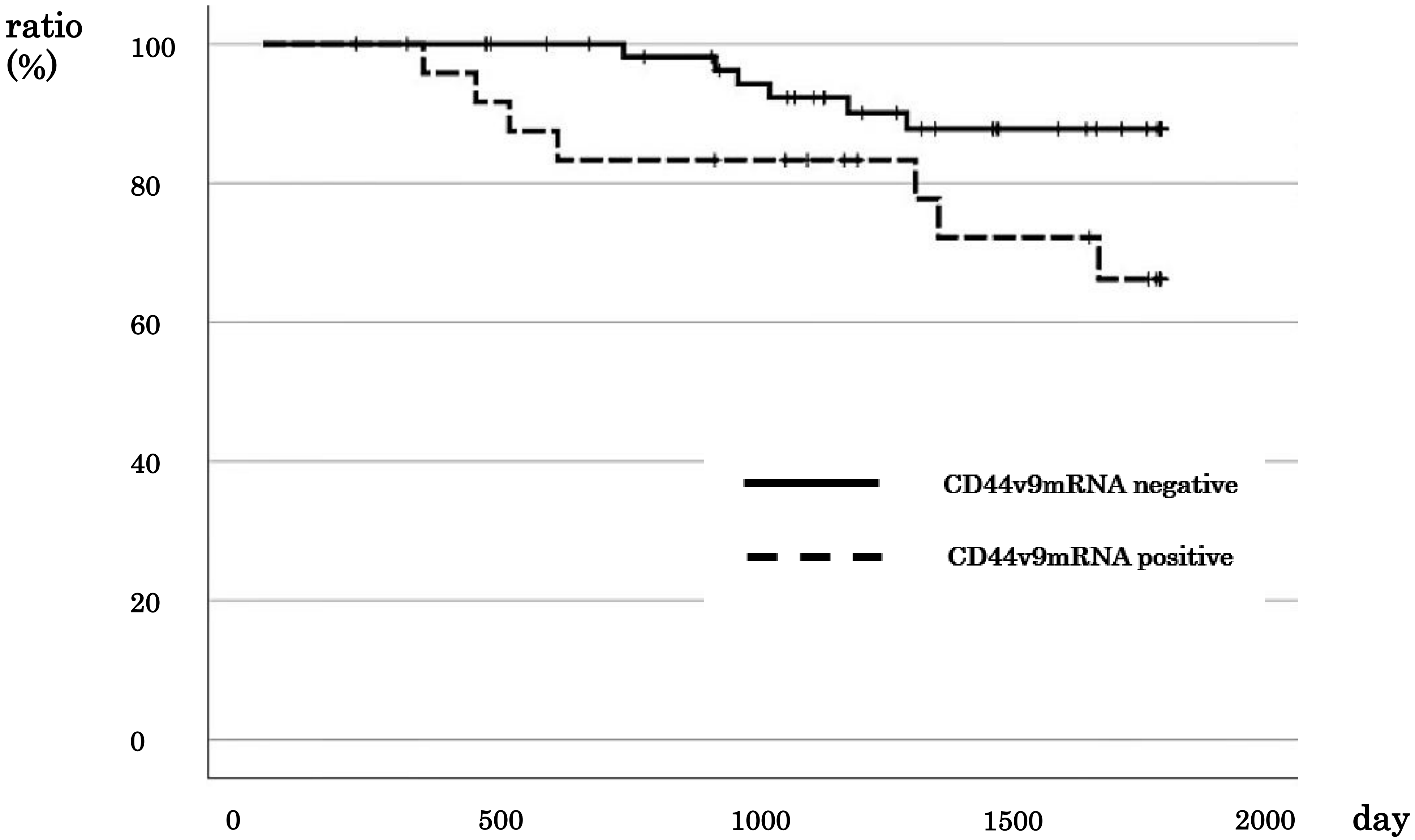

Figure S1c. Relationship between *CD44v9* mRNA expression and survival rate in patients with stage III of colorectal cancer.

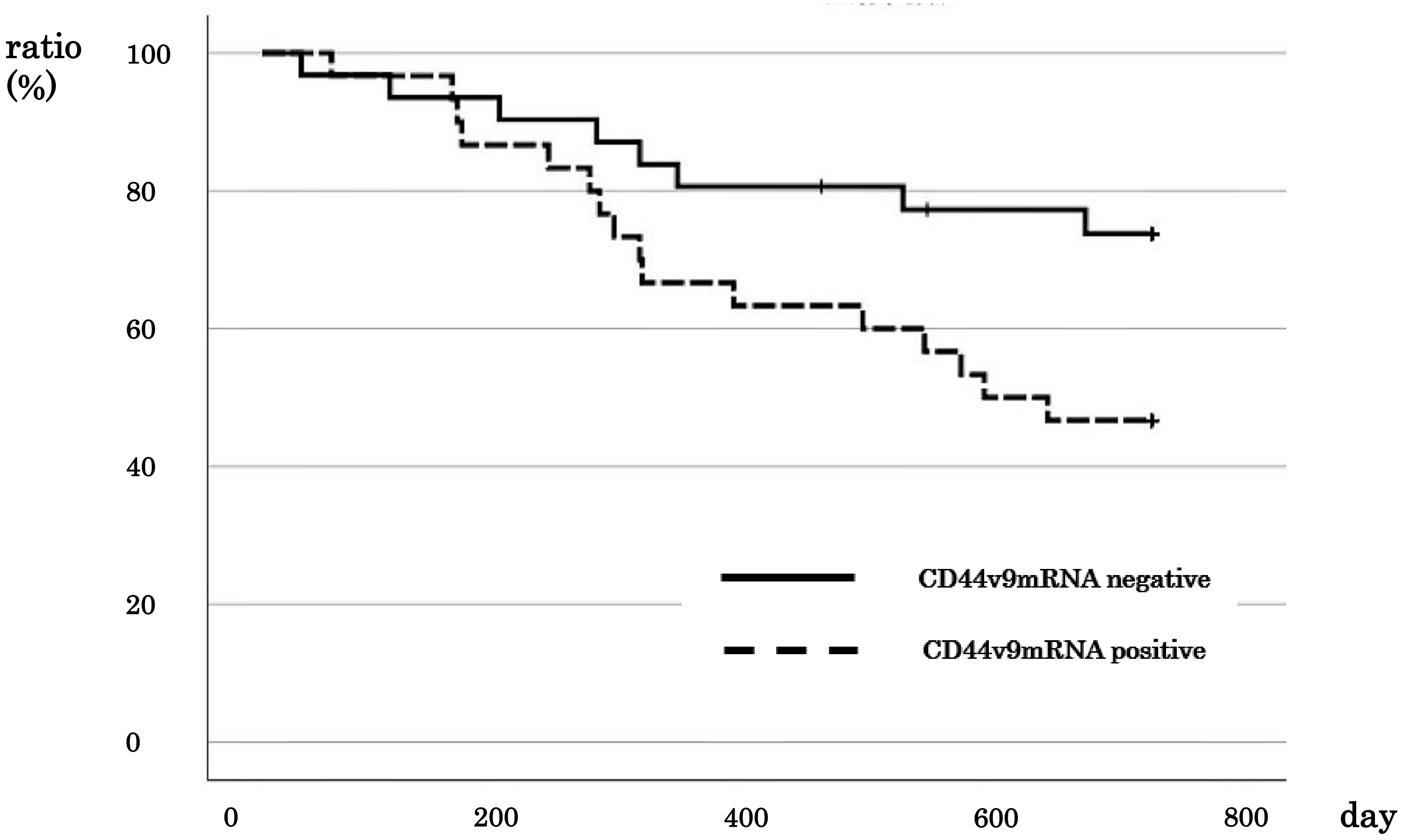

Figure S1d. Relationship between *CD44v9* mRNA expression and survival rate in patients with stage IV of colorectal cancer.

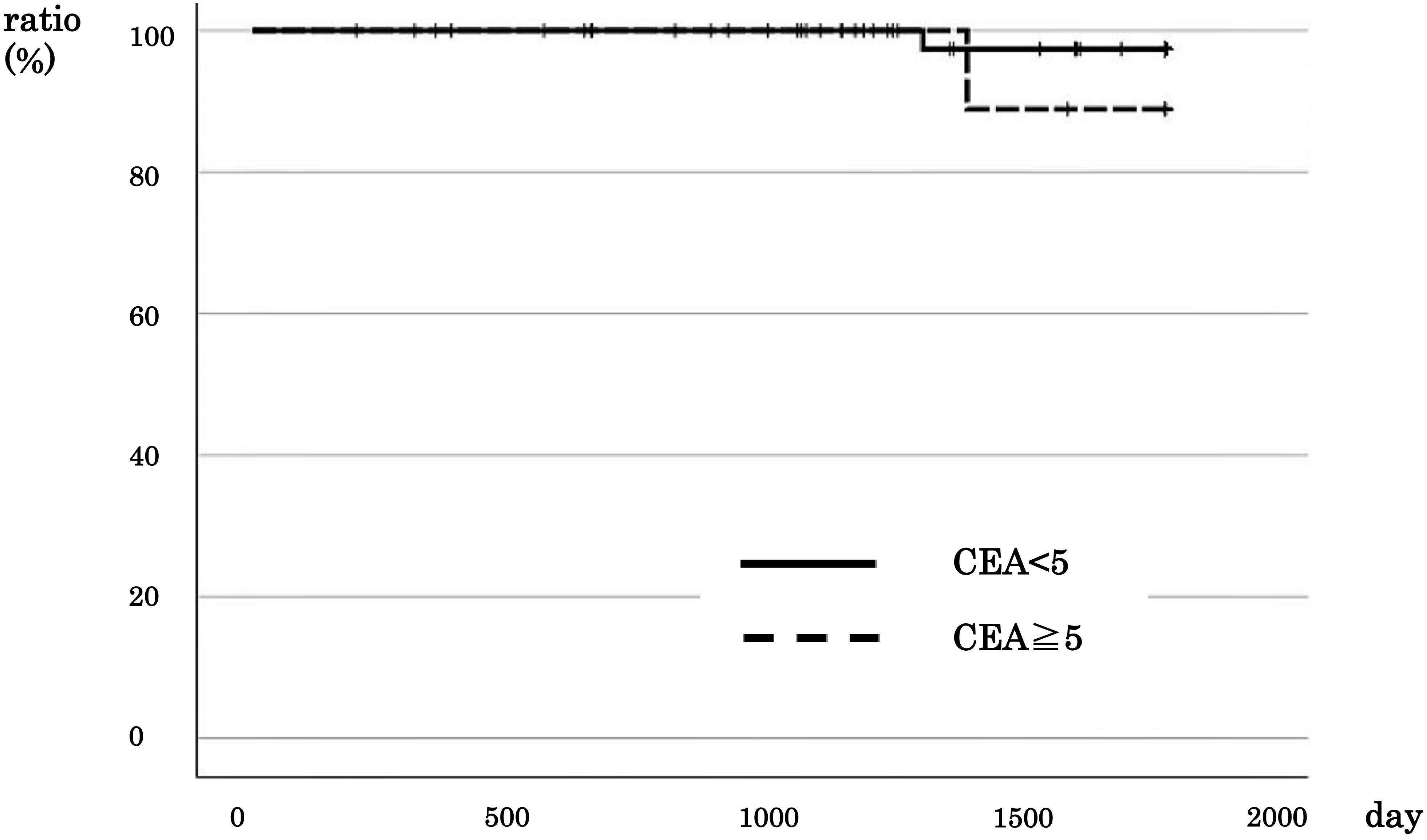

Figure S2a. Relationship between CEA values and survival rate in patients with stage I of colorectal cancer.

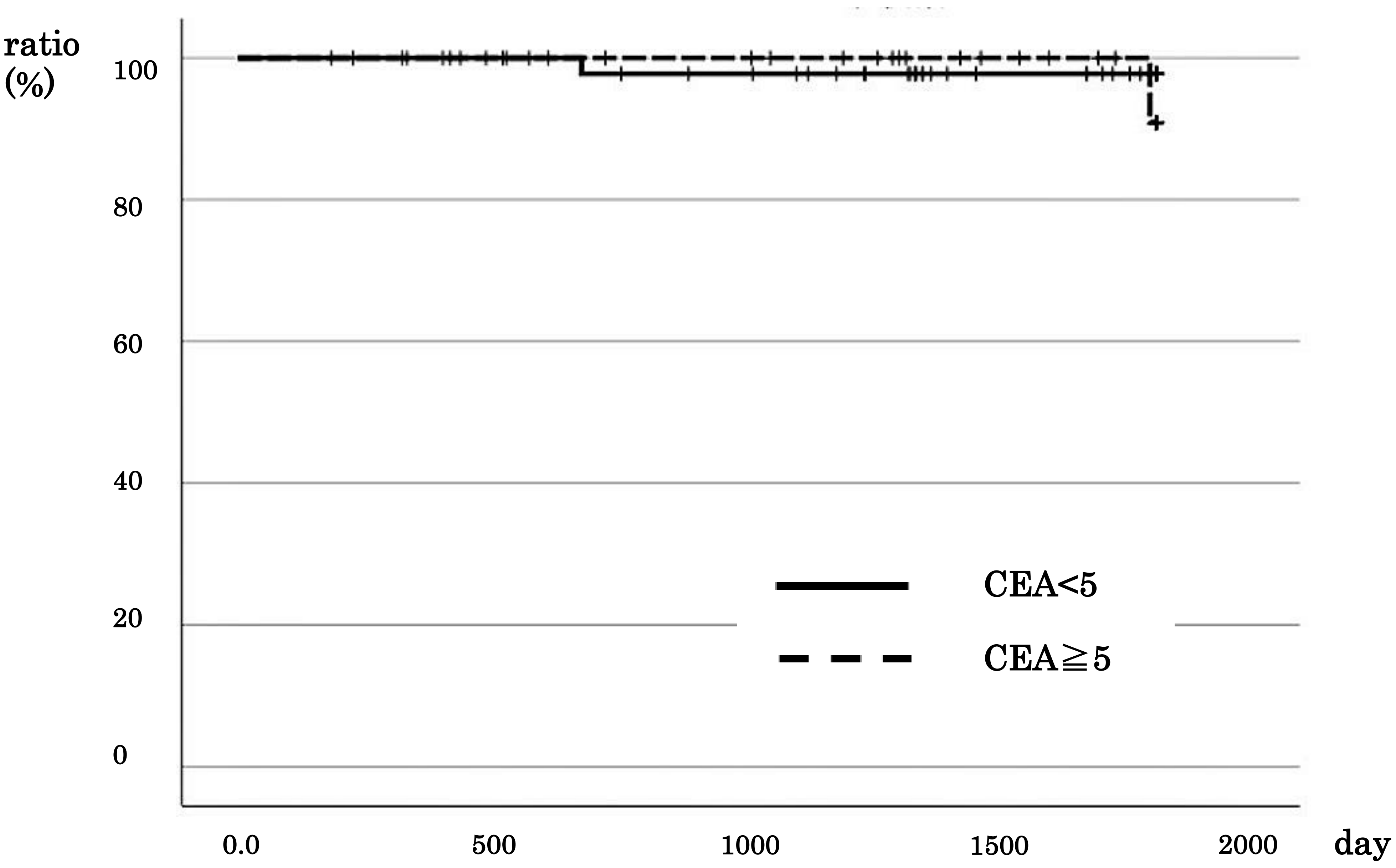

Figure S2b. Relationship between CEA values and survival rate in patients with stage II of colorectal cancer.

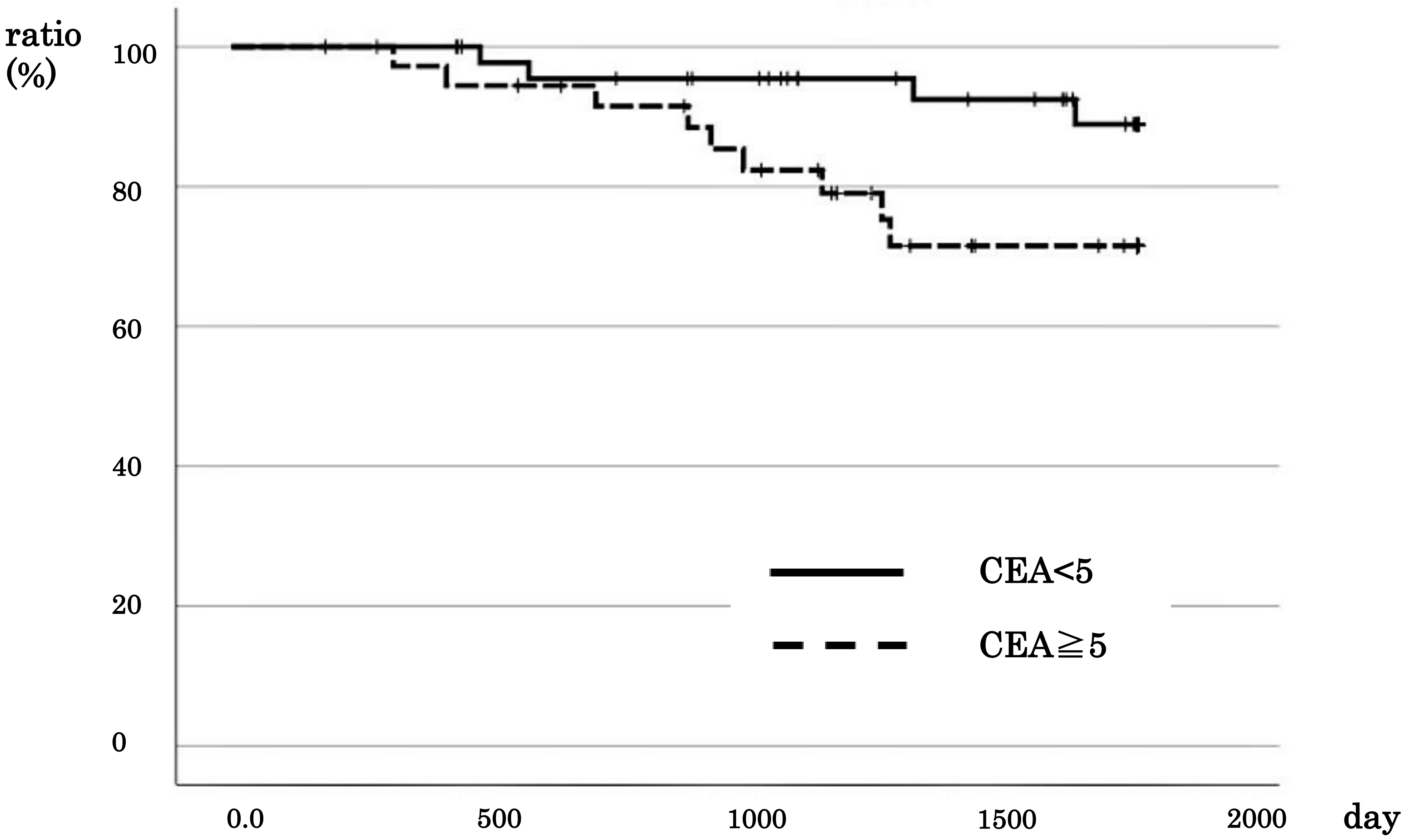

Figure S2c. Relationship between CEA values and survival rate in patients with stage III of colorectal cancer.

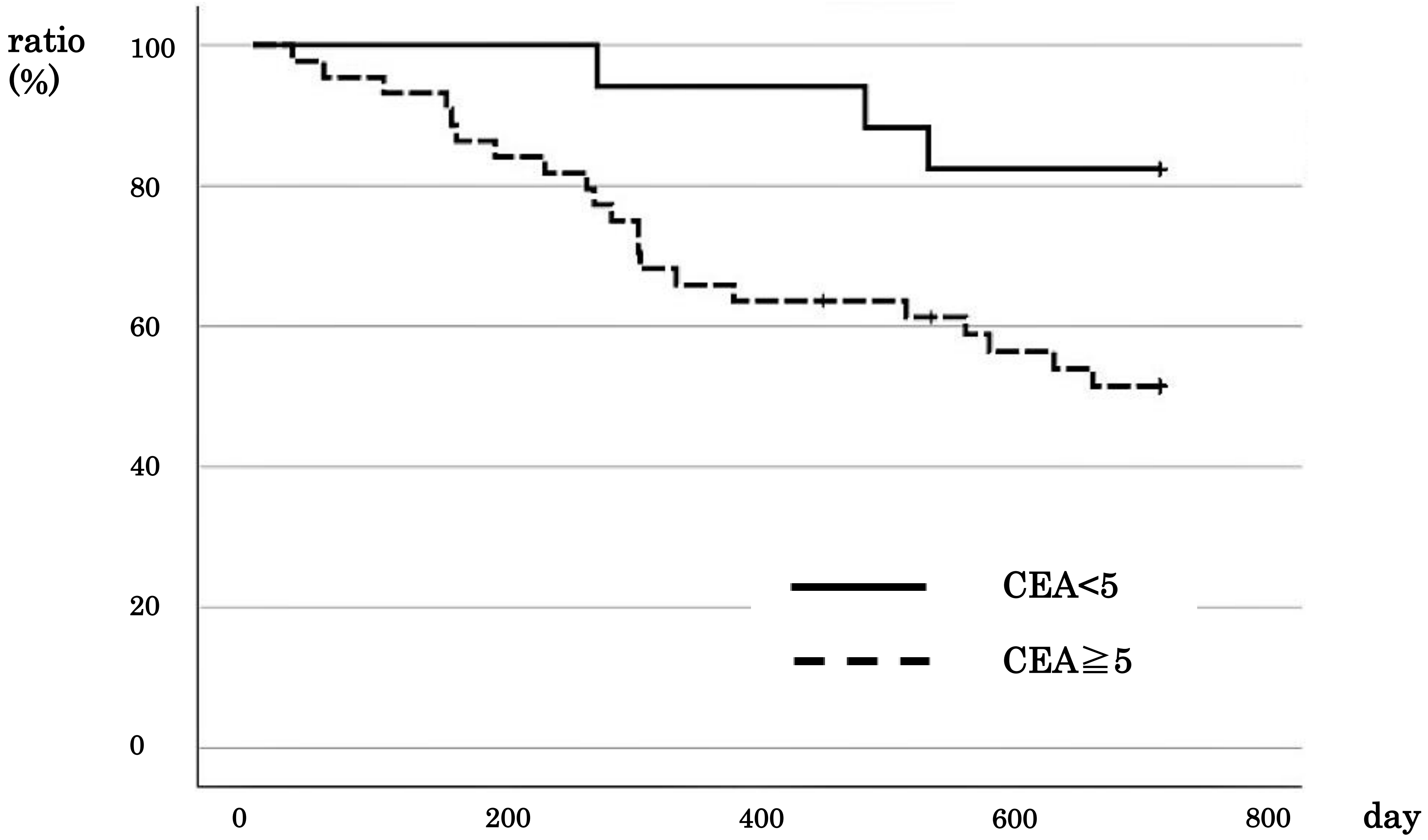

Figure S2d. Relationship between CEA values and survival rate in patients with stage IV of colorectal cancer.

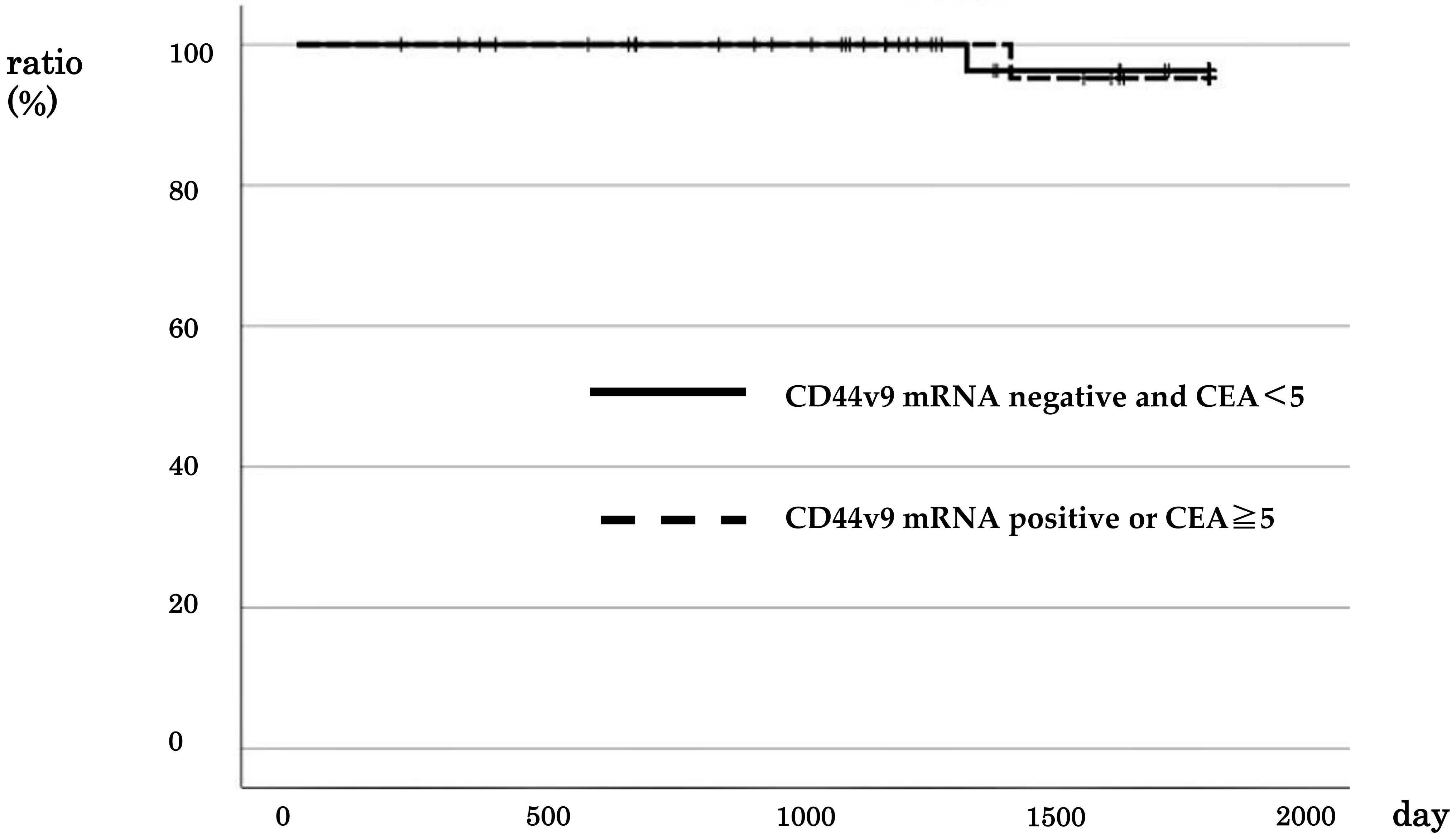

Figure S3a. Relationship between negative *CD44v9* mRNA expression and CEA < 5 and survival rate in patients with stage I of colorectal cancer.

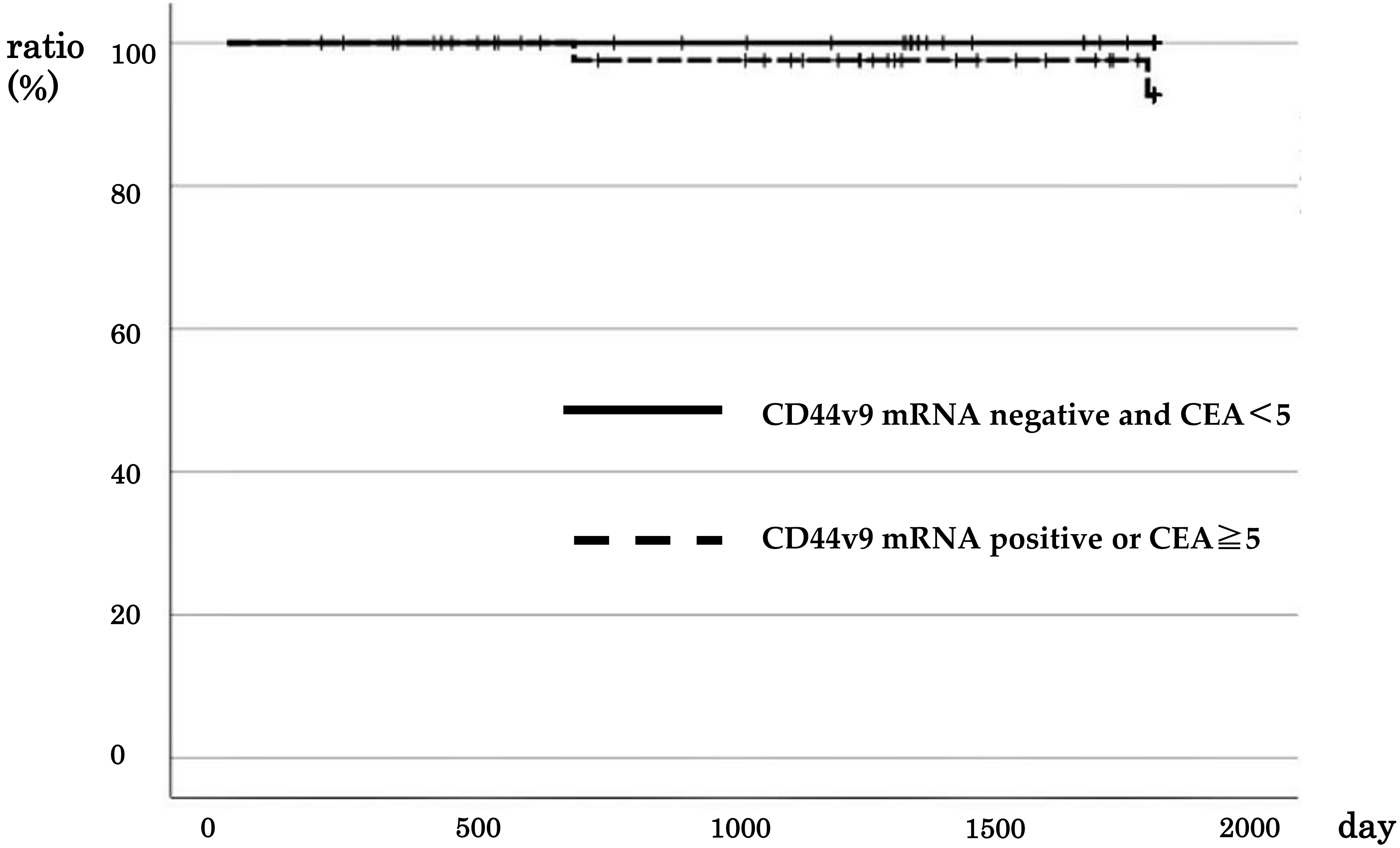

Figure S3b. Relationship between negative *CD44v9* mRNA expression and CEA < 5 and survival rate in patients with stage II of colorectal cancer.

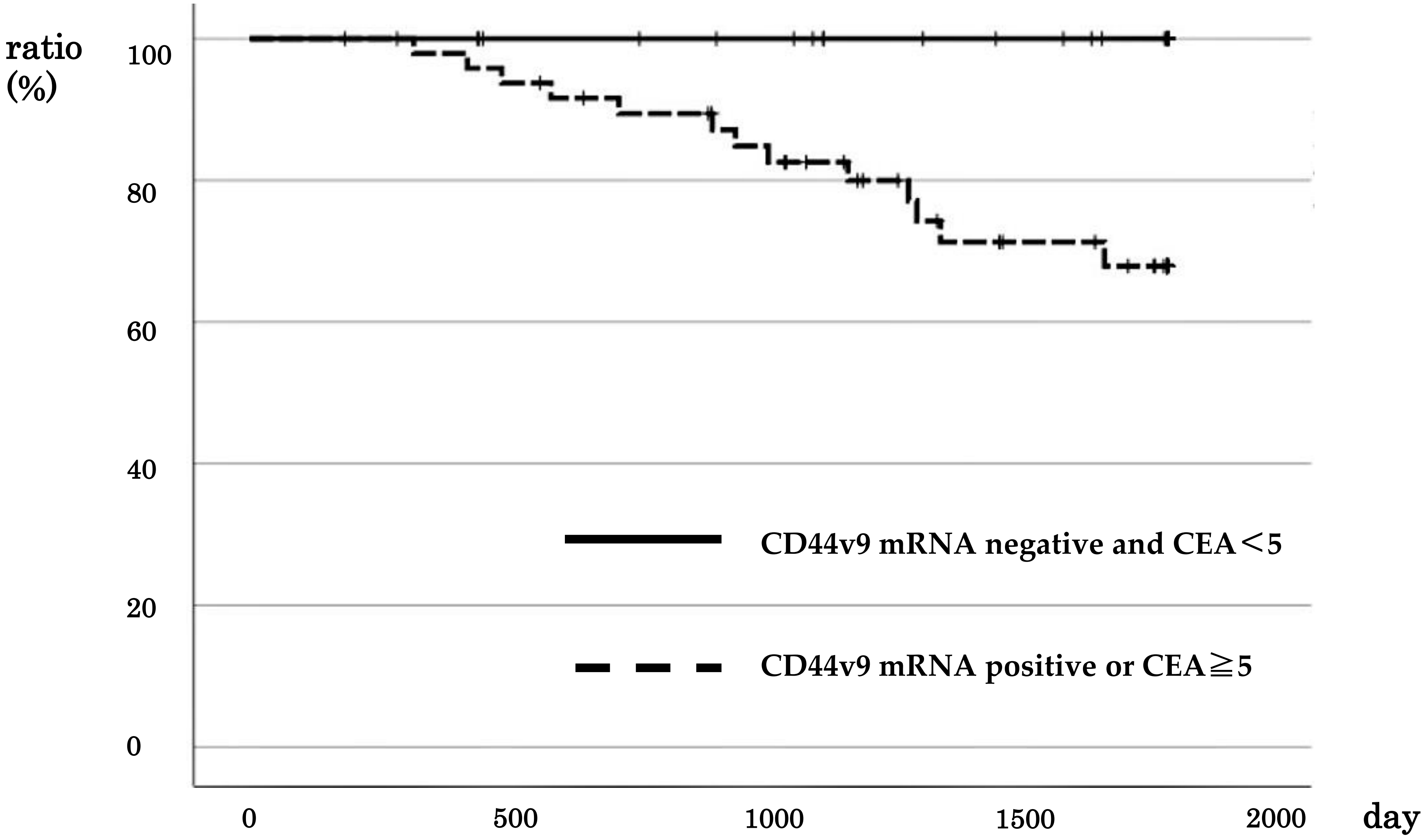

Figure S3c. Relationship between negative *CD44v9* mRNA expression and CEA < 5 and survival rate in patients with stage III of colorectal cancer.

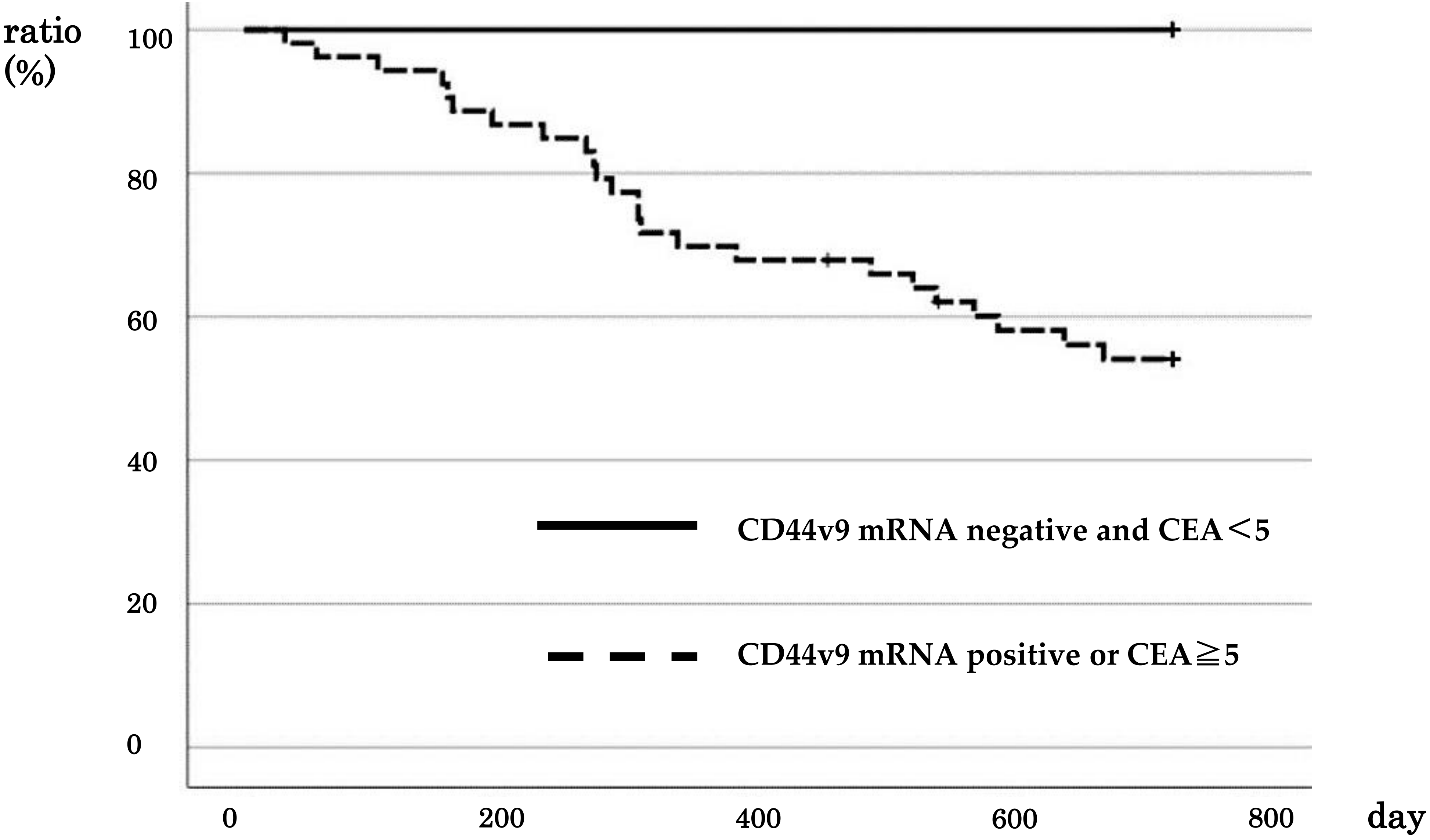

Figure S3d. Relationship between negative *CD44v9* mRNA expression and CEA < 5 and survival rate in patients with stage IV of colorectal cancer.

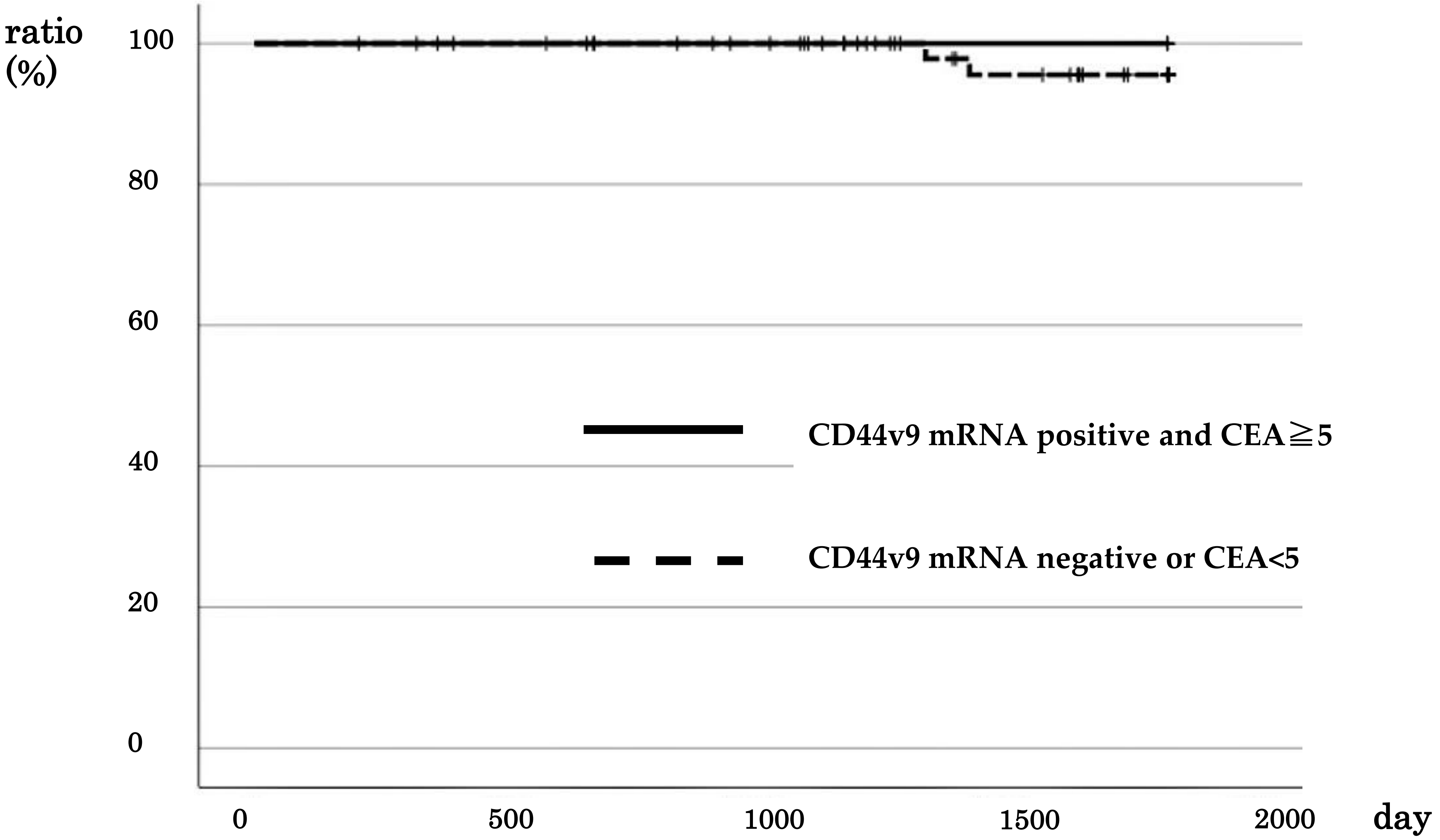

Figure S4a. Relationship between positive *CD44v9* mRNA expression and CEA≧ 5 ng/mL and survival rate patients with stage I of colorectal cancer.

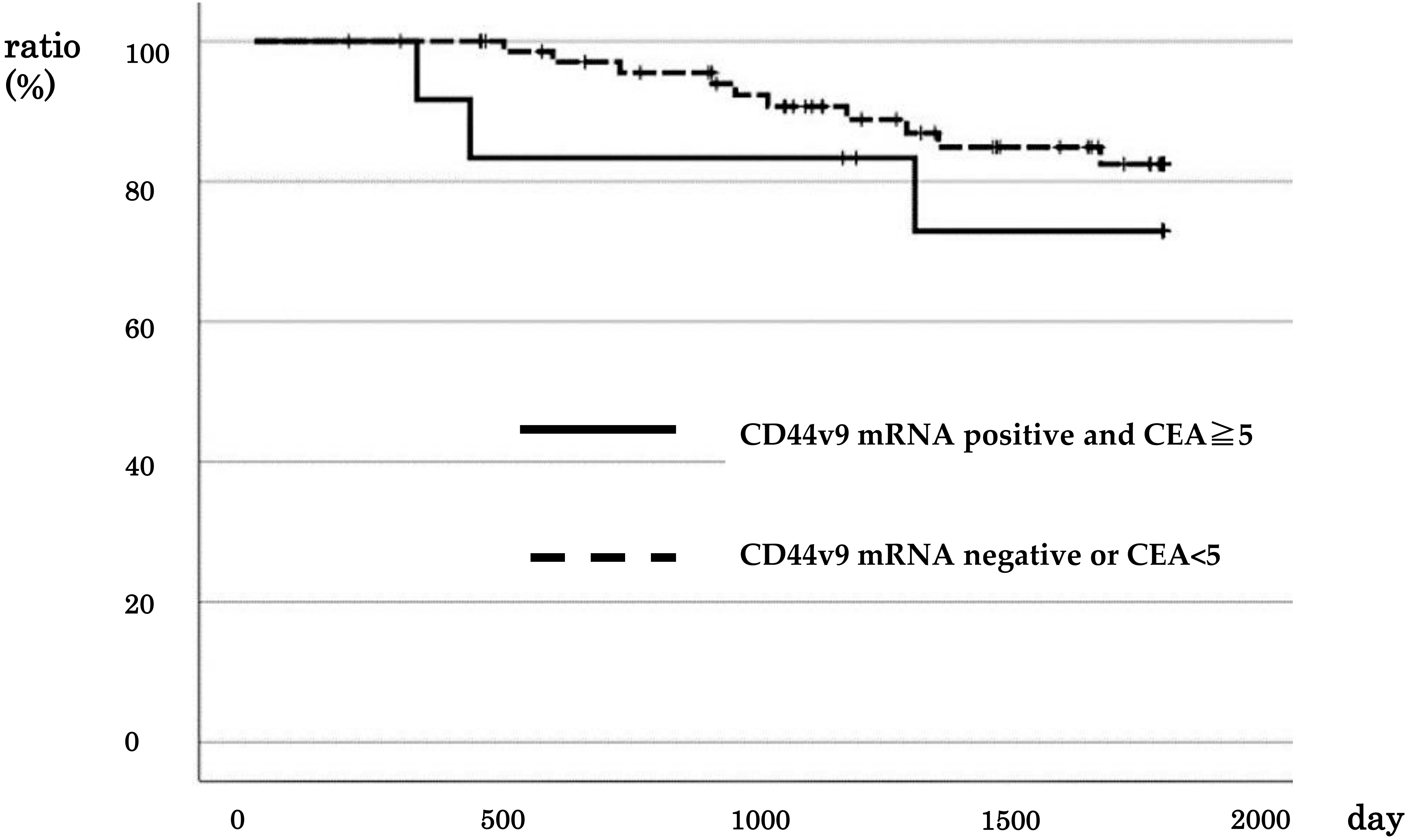

Figure S4b. Relationship between positive *CD44v9* mRNA expression and CEA≧ 5 ng/mL and survival rate patients with stage III of colorectal cancer.

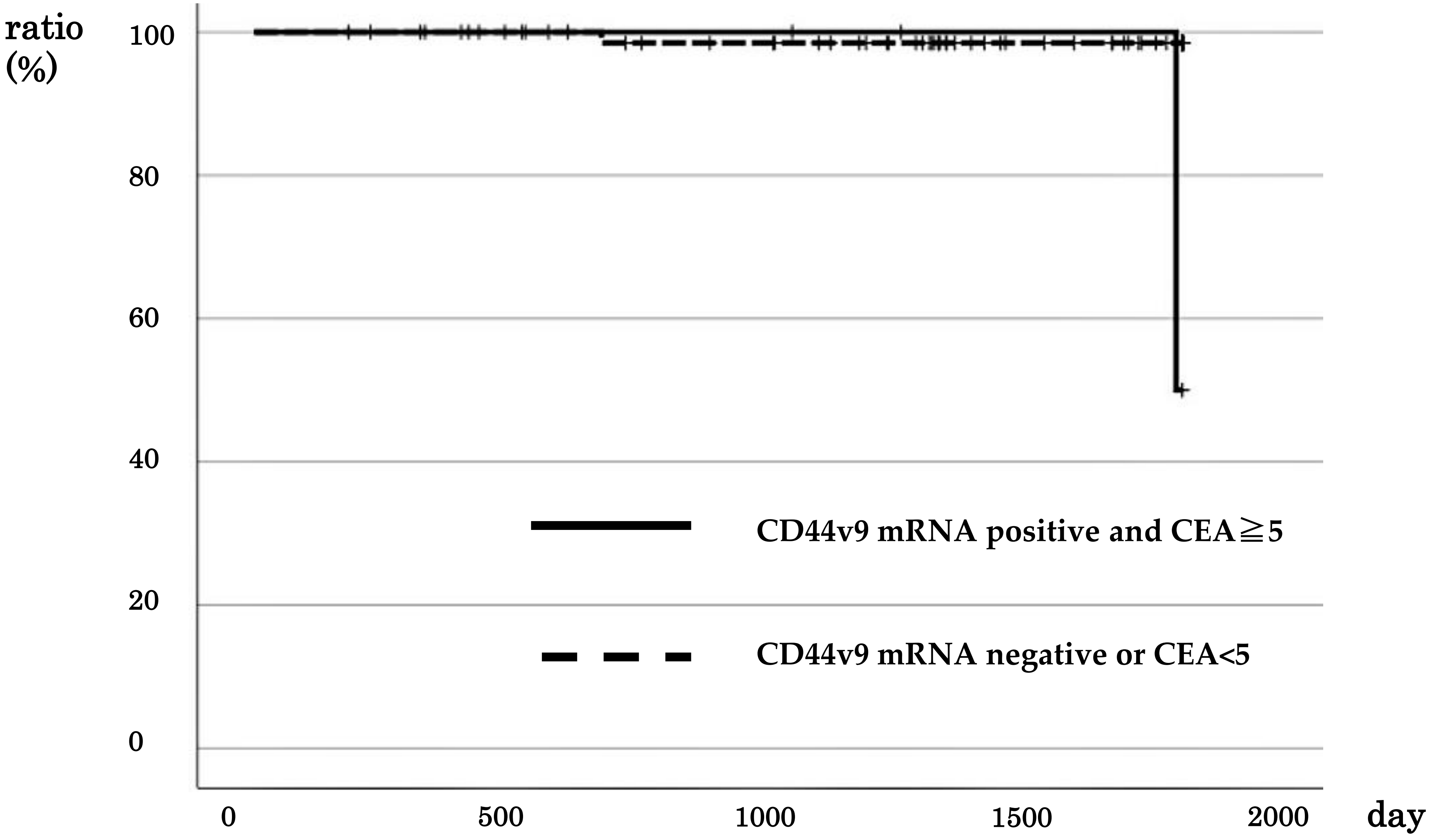

Figure S4c. Relationship between positive *CD44v9* mRNA expression and CEA≧ 5 ng/mL and survival rate patients with stage II of colorectal cancer.

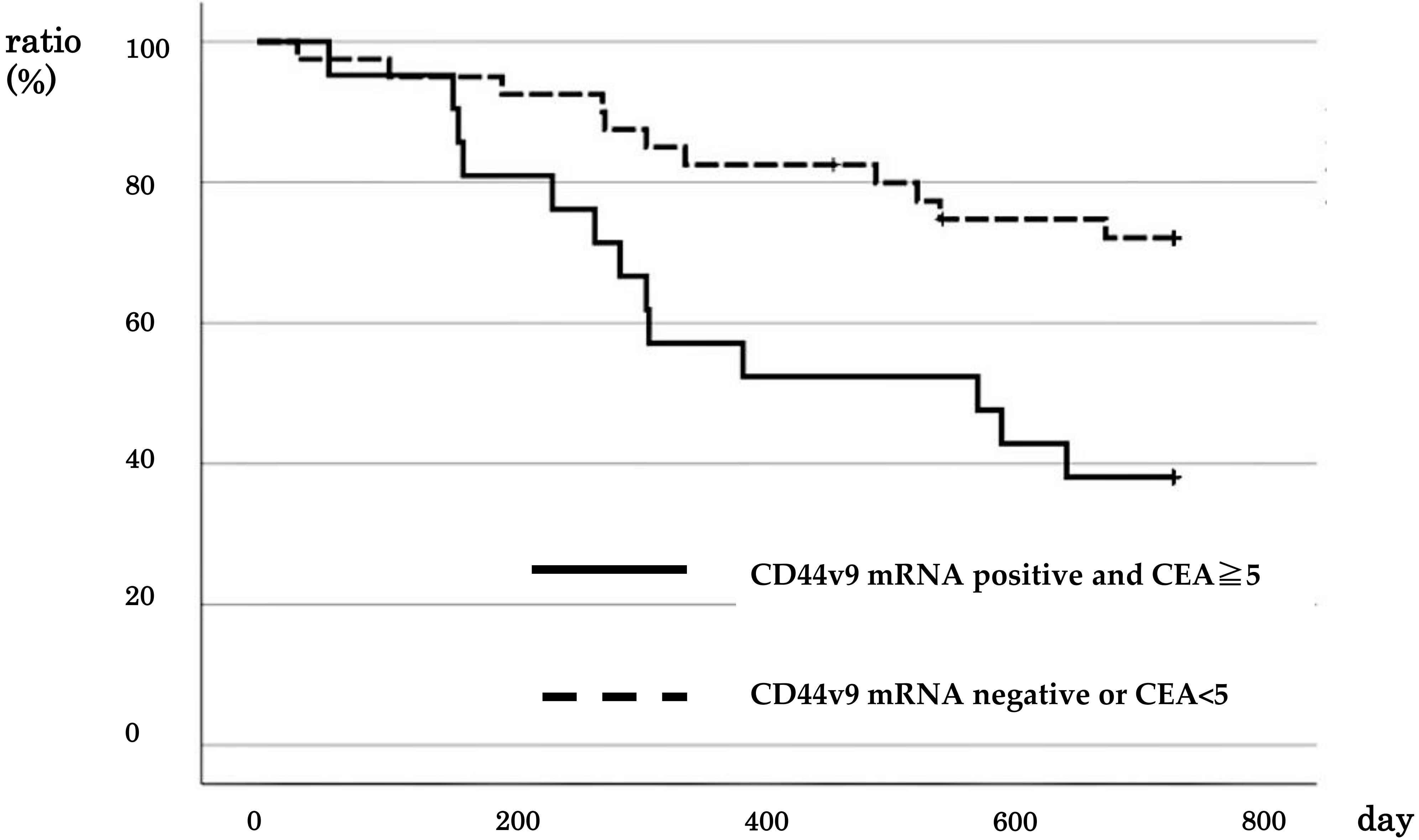

Figure S4d. Relationship between positive *CD44v9* mRNA expression and CEA≧ 5 ng/mL and survival rate patients with stage IV of colorectal cancer.
