# Supplementary figures and images for: Presence of CD44v9-Expressing Cancer Stem Cells in Circulating Tumor Cells and Effects of Carcinoembryonic Antigen Levels on the Prognosis of Colorectal Cancer
Source: Cancers (Basel). 2024 Apr 19;16(8):1556. doi: 10.3390/cancers16081556 (PMC11048819; doi:10.3390/cancers16081556)

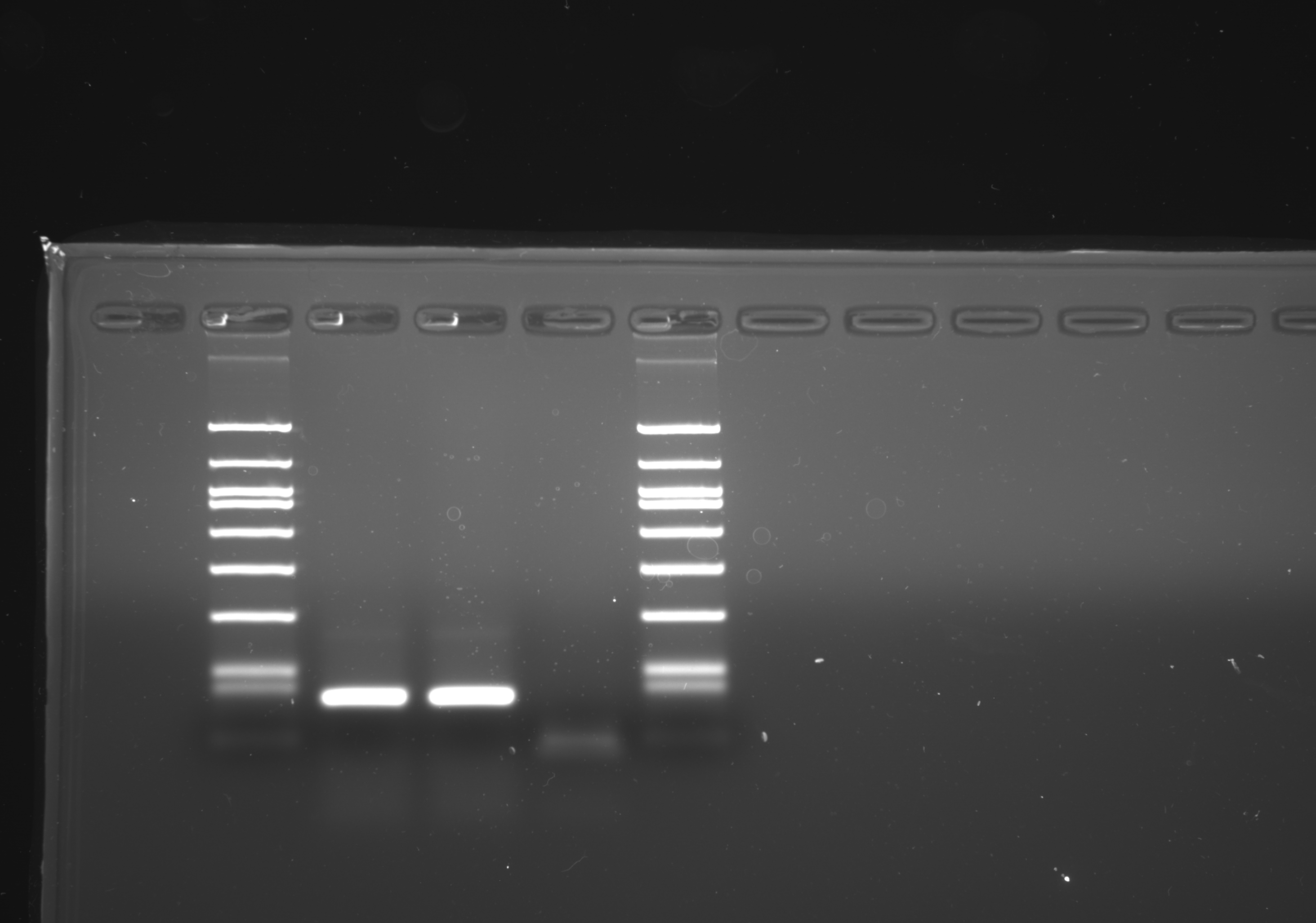

Supplement: Supplementary file 1 [file cancers-16-01556-s001.zip › Figure S5 Original western blots.tif]
